# Migrations and habitat use of the smooth hammerhead shark (*Sphyrna zygaena*) in the Atlantic Ocean

**DOI:** 10.1371/journal.pone.0198664

**Published:** 2018-06-06

**Authors:** Catarina C. Santos, Rui Coelho

**Affiliations:** 1 Portuguese Institute for the Ocean and Atmosphere, I.P., Olhão, Portugal; 2 Center of Marine Sciences (CCMAR), University of Algarve, Faro, Portugal; Hellenic Centre for Marine Research, GREECE

## Abstract

The smooth hammerhead shark, *Sphyrna zygaena*, is a cosmopolitan semipelagic shark captured as bycatch in pelagic oceanic fisheries, especially pelagic longlines targeting swordfish and/or tunas. From 2012 to 2016, eight smooth hammerheads were tagged with Pop-up Satellite Archival Tags in the inter-tropical region of the Northeast Atlantic Ocean, with successful transmissions received from seven tags (total of 319 tracking days). Results confirmed the smooth hammerhead is a highly mobile species, as the longest migration ever documented for this species (> 6600 km) was recorded. An absence of a diel vertical movement behavior was noted, with the sharks spending most of their time at surface waters (0–50 m) above 23°C. The operating depth of the pelagic longline gear was measured with Minilog Temperature and Depth Recorders, and the overlap with the species vertical distribution was calculated. The overlap is taking place mainly during the night and is higher for juveniles (~40% of overlap time). The novel information presented can now be used to contribute to the provision of sustainable management tools and serve as input for Ecological Risk Assessments for smooth hammerheads caught in Atlantic pelagic longline fisheries.

## Introduction

With the rapid expansion of fishing fleets and the increasing exploitation of the open ocean, many marine predators have experienced a decline over the past decades [1, 2]. Among the impacted species, large elasmobranchs (including sharks) have been of particular concern [3]. Pelagic sharks are caught by a variety of fishing gear and are common as bycatch of pelagic longline fleets targeting mainly swordfish (*Xiphias gladius*) and tunas (*Thunnus* spp.) [4–8]. Since predators play a major role in marine communities’ structure and function, widespread decline of sharks across the world’s oceans are expected to strongly influence the equilibrium of marine ecosystems [6, 9]. Therefore, understanding habitat use and ecology of sharks is crucial to evaluate the impacts of fishing on them and throughout the food web. Additionally, for many pelagic shark species, important information on their life history and ecology is still missing, as well as survey or reliable catch data, which hinders higher level scientific-based management advice.

The smooth hammerhead shark, *Sphyrna zygaena*, is a semipelagic shark distributed worldwide in temperate and tropical waters, most commonly in waters shallower than 20 m along the water column [4, 10]. As most elasmobranchs, the smooth hammerhead is characterized by having slow-growth, late maturity and relatively low fecundity, which makes the species relatively vulnerable to fishing mortality [4, 6, 11–13]. Cortés et al. [12] conducted an Ecological Risk Assessment (ERA) and concluded that the smooth hammerhead had a medium vulnerability specific to pelagic longline fisheries in the Atlantic Ocean. ERAs are assessment tools that can be used to evaluate the overall vulnerability of a stock to overexploitation, taking into account its biological productivity and susceptibility to a fishery [14]. In this way, out of the 20 assessed shark stocks, smooth hammerheads ranked 13^th^ in terms of their overall vulnerability to the pelagic longline fisheries. Furthermore, the study concluded that the quality and extent of the information available for the smooth hammerhead were considerably lower than those available for other species, highlighting the need for better and improved biological and ecological data for this species [12].

The smooth hammerhead is listed as “Vulnerable” by the International Union for the Conservation of Nature (IUCN) [10] and was given international level protection in terms of trade under Appendix II of the Convention for International Trade in Endangered Species (CITES) [15]. Also, sustainability concerns regarding this species led the International Commission for the Conservation of Atlantic Tunas (ICCAT) to issue several management regulations concerning the conservation of the smooth hammerhead in the Atlantic Ocean. Specifically, fishing vessels are prohibited from “retaining onboard, transshipping, landing, storing, selling, or offering for sale any part or whole carcass of hammerhead sharks of the family Sphyrnidae (except for the *Sphyrna tiburo*), taken in the Convention area in association with ICCAT fisheries" [16]. Additionally, ICCAT has specific requests aimed to fill knowledge gaps for this species (see paragraph 5 of ICCAT Recommendation 10–08, [16]), Despite being occasionally captured as bycatch by industrial longline fleets targeting swordfish and tunas in the Atlantic Ocean—though in much lower numbers than the considerably more common blue shark (*Prionace glauca*) and shortfin mako shark (*Isurus oxyrinchus*) [5, 14]-, very limited information is currently available on the smooth hammerhead life history parameters, movement patterns, essential habitat and population dynamics [13, 17]. The situation is aggravated by likely misidentification problems within the different hammerhead shark species [8].

Studying fishes in their natural environment is a difficult task, however tracking techniques and satellite tagging have experienced rapid development over the past decades, providing scientists the opportunity to improve the knowledge on spatial ecology of marine predators, like pelagic sharks [18–22], tunas [23] and swordfish [24]. Apart from providing estimates of fish position (through light level data), current-generation Pop-up Satellite Archival Tags (PSATs) also collect and record pressure (depth) and water temperature data at set intervals of a few seconds to several hours [25]. After being programmed to collect information for a predetermined amount of time, the PSAT releases from its host and floats to the surface where it sends its broadcast of data to the ARGOS satellite-based system [25, 26]. The data transmitted from the satellite tags can then be used to understand distribution ranges, movement patterns, and calculate overlaps between the vertical habitat use and depths of hooks of pelagic longline fishing gear. Such information can therefore improve the knowledge needed to provide scientific recommendations for optimal species management and conservation.

Previous studies using satellite telemetry on smooth hammerhead sharks are limited and none were carried out in the Atlantic. In the Pacific, one smooth hammerhead shark tagged off San Clemente Island, California was documented to travel south to the central Baja Peninsula and then return north to Ventura, California, travelling a total distance of more than 1600 km [27]. More recently, five smooth hammerheads were tagged in northern New Zealand, with information from three tags successfully transmitted. Besides noting the ability of the species to travel significant distances (> 150 km), the study also revealed that smooth hammerhead sharks generally occurred mostly in surface waters (0–60 m) [28]. For other hammerhead species, particularly for the scalloped hammerhead (*Sphyrna lewini*), some previous studies using PSATs were carried out in the Pacific and Atlantic Oceans (Gulf of Mexico) that described vertical and horizontal movement patterns [29–32]. These studies indicated the scalloped hammerhead is mainly a coastal species, tolerant to a wide range of temperatures (4–28°C). Contrary to the smooth hammerhead, the scalloped hammerhead showed more consistent diel movement pattern, while similarly to the smooth hammerhead deep diving behavior (in this case > 980 m) was documented.

Given the limited data currently available on the habitat use and vulnerability to fisheries for the smooth hammerhead shark, and the need of such information to provide informed advice for management and conservation of the species, the objectives of this study were 1) to improve the knowledge of movement patterns in the inter-tropical Atlantic Ocean, 2) to investigate vertical habitat use in terms of diel movements and 3) to calculate the overlap between the vertical habitat use and the depth of hooks of pelagic longline fishing gear, specifically from surface drifting longlines targeting swordfish.

## Materials and methods

### Tagging procedure

A total of 8 PSATs were used. Tagging took place in international waters of the inter-tropical region of the Northeast Atlantic Ocean, between September 2012 and August 2016, and was carried out by scientific fisheries observers from the Portuguese Institute for the Ocean and Atmosphere, I.P. (IPMA), which is the National Institute responsible for collecting and reporting data on incidental catches of this species to ICCAT. Tagging was conducted onboard vessels from the Portuguese pelagic longline fleet, which were licensed by the Portuguese Fisheries Administration (DGRM—Direcção Geral de Recursos Naturais, Segurança e Serviços Marítimos) and ICCAT to operate with pelagic longline gear in that region. The study was presented and received the necessary endorsement by the ICCAT SCRS (Standing Committee on Research and Statistics). The PSAT deployment was opportunistic when smooth hammerheads were captured during the regular pelagic longline fishing operations, using J-style hooks and wire leaders. The tagging protocol adopted by IPMA was followed, with the smooth hammerhead specimens tagged and released immediately upon capture, without anesthesia, by cutting the gangion line as close as possible to the mouth. Sharks were either hoisted alongside the vessel or brought on board for tagging. In addition, the animals were sexed and measured for fork length (FL). Date and time were recorded, and the geographic tagging location (latitude and longitude) was determined by Global Positioning System (GPS). The entire tagging operation lasted a maximum of 2 minutes, and did not produce any additional injuries or damage to the specimens.

The PSATs were rigged with wire leaders (aprox. 15 cm in length) secured with stainless steel crimps and encased in surgical silicone tubing. The crimps were at a distance from the tag sufficient to prevent any accidental contact with the PSATs detachment mechanism and were covered with silicone tubing. An umbrella-type nylon dart [33] was used to insert the tag laterally to the dorsal musculature below the first dorsal fin, using the methodology described by Howey-Jordan et al. [34]. Before tag attachment, PSATs were tested for accurate data collection, and were programmed to record information for periods between 31 and 150 days (Table 1).

**Table 1:** Characteristics of tagged smooth hammerhead sharks and PSATs.

ID	Tag Model	Size (FL, cm)	Sex	Maturation state	Tagging date	Planned duration (days)	Tracking days	Transmitted data (%)
113781	MTI Standard	170	Male	Adult	3-Sep-2012	90	28	100
113784	MTI Standard	170	Female	Juvenile	11-Oct-2012	120		
127998	MTI X-tag-HR	175	Female	Juvenile	1-Oct-2013	31	31	56
127999	MTI X-tag-HR	130	Female	Juvenile	17-Nov-2013	31	31	7
136856	WC MiniPAT	123	Female	Juvenile	27-Jul-2014	120	6	44
136143	MTI X-tag	180	Male	Adult	18-Sep-2014	60	6	100
160917	WC MiniPAT	180	Female	Juvenile	27-May-2016	150	67	74
162392	WC MiniPAT	205	Female	Adult	2-Aug-2016	150	150	78

Characteristics of tagged smooth hammerhead sharks, *Sphyrna zygaena*, and PSATs used in this study, with information on specimen size, sex, maturation state, tag type, planned duration, effective tracking days and % of transmitted data. FL = fork length.

Four models of PSATs were used: Standard, X-tags and high rate (MTI-HR) X-tags manufactured by Microwave Telemetry, Inc. (MTI) and MiniPAT tags built by Wildlife Computers (WC). Tags specifications are presented in Table 2.

**Table 2:** Tags specificafions.

	Tag Model			
Specifications	MTI Standard	MTI X-tag	MTI X-tag-HR	WC MiniPAT
Sensors	Depth, Temperature, Light	Depth, Temperature, Light	Depth, Temperature, Light	Depth, Temperature, Light
Depth sensor range	0–1296 m	0–1296 m	0–1296 m	0–1700 m
Depth sensor resolution	5.4 m	0.34–5.5 m	0.34–1.34 m	0.5 m
Temperature sensor range	-4-40°C	-4-40°C	-4-40°C	-20-50°C
Temperature sensor resolution	0.16–0.23°C	0.16–0.23°C	0.16–0.23°C	0.05°C
Transmission interval	15 min.	15 min.	5 min.	10 min.

Specifications of the PSATs used in this study, with information on depth and temperature sensors and data transmission time interval.

### Depth of longline gear operation

In order to characterize the depth of pelagic longline operations, Minilog Temperature and Depth Recorders (TDRs) made by Vemco (Bedford, Nova Scotia, Canada) were deployed on 60 fishing sets. Six TDRs were used per fishing set and were programmed to record data at 1 min interval, with a resolution of 1.2 m. TDRs were attached adjacent to hooks and placed on all hooks between floats.

The fishing sets were carried out following the general practices of the European shallow pelagic longline fleet that targets mainly swordfish, with gear setting typically starting in the late afternoon, and retrieval commencing at dawn of the next morning. Details of the fishing gear are described in Coelho et al. [21], consisting of a standard US-style polyamide monofilament mainline, with five branch lines (~ 18 m long and with a J-style hook in the terminal tackle) between floats. Two different size options for the float line are typically used by this fleet: either 12 m or 16 m. Consequently, this variability of the fleet fishing strategy was considered in the study design, with the TDRs equally deployed on sections using both sizes of float lines.

### Data analysis

Habitat use was investigated by calculating the percentage of time-at-depth and time-at-temperature, and was separately analyzed for daytime and nighttime. Sunset and sunrise were calculated using library “RAtmosphere” in R [35], and took into account the date (Julian day), latitude and longitude [36]. Habitat use was also analyzed separately for juvenile and adult specimens. The definition of juvenile and adult stages was based on the Compagno [4] values of size at first maturity of 210 and 240 cm total length (TL) for males and females, respectively. Because size data in our study refer to FL, we used the conversion factor from Mas et al. [37] to convert sizes from TL into FL: FL = 0.78 × TL.

Time-at-depth and time-at-temperature data were aggregated into 10 m and 1°C bins, respectively, based on the above analyses. These data were subsequently expressed as a fraction of the total time of observation for each shark, and the fractional data bins averaged across all sharks within each category. The depth and temperature data were tested for normality with Kolmogorov-Smirnov tests with Lilliefors correction [38] and for homogeneity of variances with Levene tests [39]. Given the lack of normality in the data and heterogeneity of variances, time-at-depth and time-at-temperature were compared between the daily period (daytime *vs*. nighttime) and maturity stage (adults *vs*. juveniles) with nonparametric k-sample permutation tests [40], using library “perm” in R [41]. For this, a Monte Carlo approach was used with the data randomized and re-sampled 9999 times to build the expected distribution of the differences under a random distribution, which was then used to determine the significance of the differences in the time-at-depth and time-at-temperature for the sample.

Geographic positions at tagging were determined by GPS, while the pop-up locations of transmitting PSATs were established as the first point of transmission with an Argos satellite. The most probable tracks between tagging and pop-up locations were calculated from PSATs light level data using astronomical algorithms provided by the tag manufacturers. To improve the geolocation quality, the unscented Kalman filter state-space model incorporating a sea surface temperature field was applied [42]. This was applied using library “ukfsst” in R [43] for the Microwave Telemetry tags and with the GPE-3 software for the Wildlife Computer tags [44]. In the case of HR X-tags, light level data cannot be used for geolocation estimates because it is stored at a lower resolution than in standard rate tags. Therefore, the distances travelled by the sharks tagged with HR X-tags were measured in straight lines between the tagging and the pop-up locations.

The overlap between the habitat use and depth of fishing gear was calculated by analyzing the results from the TDRs and PSATs. The mean depth of the hooks was calculated, and the differences between hooks set with 12 m or 16 m float lines tested with permutation tests [40]. The 90% percentiles of the recorded hook depths were calculated and the depth distribution of the specimens PSAT data were overlapped with the depth distribution of the fishing gear in order to calculate the percentage of overlap time. Overlap time data were tested for normality with Kolmogorov-Smirnov tests with Lilliefors correction [38] and for homogeneity of variances with Levene tests [39]. Since overlap time data were normally distributed and variances were homogeneous, a two-way factorial analysis of variance (ANOVA) was used to test for differences in the overlap time, taking into consideration maturity stage (juvenile and adult), daily period (daytime and nighttime) and length of float lines (12 m and 16 m).

All statistical analyses for this paper were carried out with the R language [45]. Plots were created using libraries “plotrix” [46] and “ggplot2” [47]. The ANOVA was run using library "car" [48].

## Results

### Tag performance

Eight tags were deployed during this study, with data from seven tags successfully transmitted. A total of 319 tracking days were registered (Table 3).

**Table 3:** Total tracking days.

Sex	Maturity stage		Total
	Adults (N = 3)	Juveniles (N = 4)	
Females (N = 5)	150	135	285
Males (N = 2)	34		34
Total	184	135	319

Total tracking days of smooth hammerhead sharks, *Sphyrna zygaena*, per sex (males and females) and maturity stage (adults and juveniles). N is the number of tags for each sex and maturity stage.

### Horizontal movements

**Fig 1:**
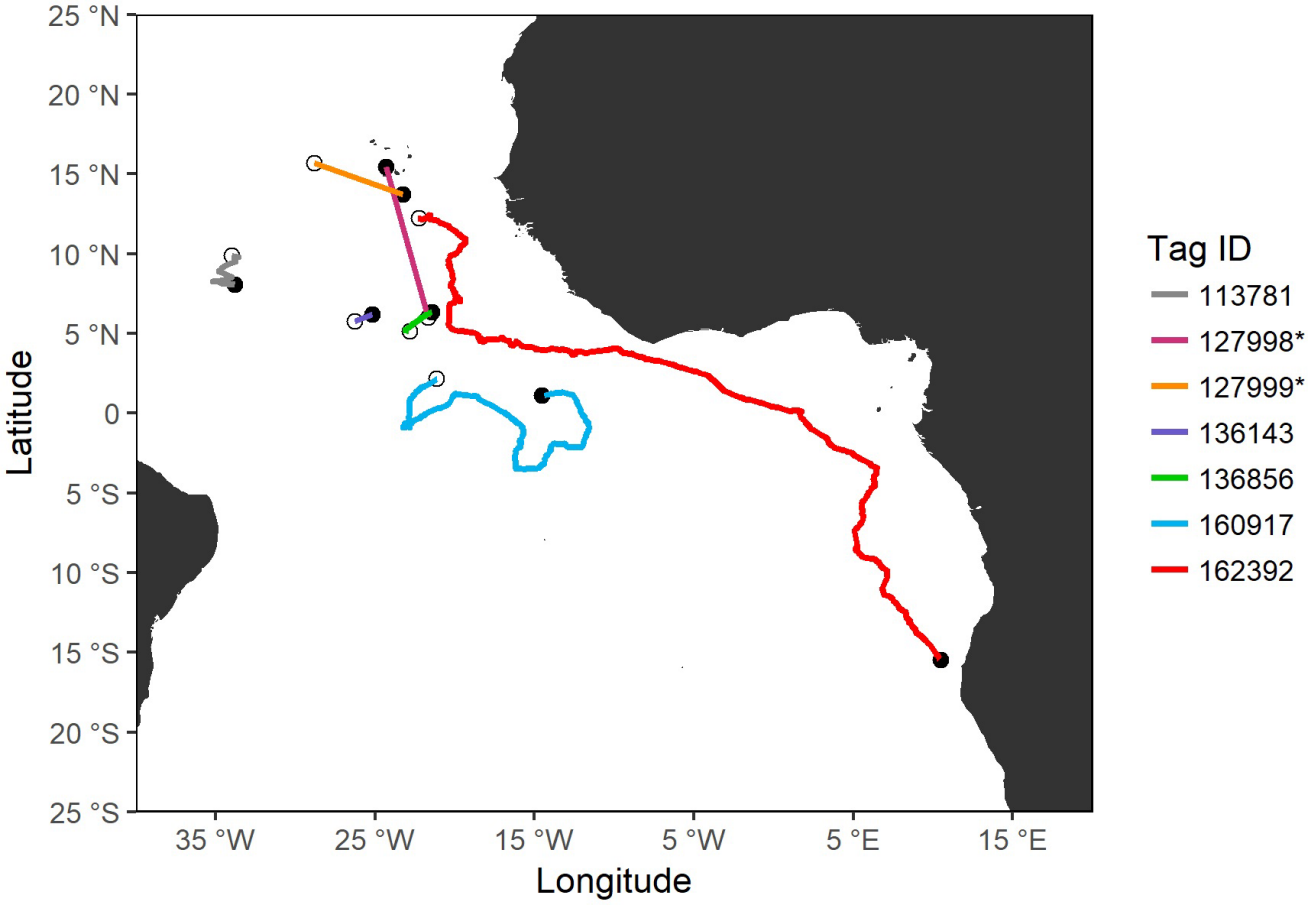
Most likely tracks of smooth hammerhead sharks. Tagging and pop-up locations of smooth hammerhead sharks, *Sphyrna zygaena*, with the respective most likely tracks estimated for each specimen. The tagging locations are represented with white circles and the pop-up locations are represented with black circles. Note that for HR X-tags (*) only strait line tracks are shown.

Estimated most likely tracks indicate that smooth hammerhead sharks spent their time in the tropical and equatorial Atlantic Ocean, swimming in both open and coastal waters (Fig 1). The distances travelled ranged from 131.7 km to 6610 km (for 6 and 150 tracking days, respectively), corresponding to an average daily distance of 33.4 km/day (Table 4).

**Table 4:** Characteristics of the tracks taken by smooth hammerhead sharks.

Shark ID	Tracking days	Distance travelled (km)	Average daily distance (km/day)
127998*	31	1092	35.2
127999*	31	636	20.5
113781	28	677	24.2
136143	6	132	22
160917	67	3037	45.3
136856	6	257	42.8
162392	150	6610	44.1

Characteristics of the tracks taken by smooth hammerhead sharks, *Sphyrna zygaena*, with information on effective tracking days, distance travelled and average daily distance. HR X-tags are marked with an asterisk (*).

Since HR X-tags don’t record the times of sunrise and sunset, the distances travelled by the sharks 127998 and 127999 (both juvenile females) were measured in straight lines between the tagging and pop-up locations. Shark 127998 moved north, while shark 127999 swam towards southeast. Both these sharks seem to have migrated inshore towards Cape Verde archipelago coastal waters. During their movements, shark 113781 (adult male) and shark 160917 (juvenile female) followed an oscillatory track heading south and east, respectively. Shark 136143 (adult male) moved northeast and shark 136856 (juvenile female) swam in a steady northeasterly direction. Finally, shark 162392 (adult female) travelled towards southeast, along the African west coast. This shark’s course represented a trans-equatorial migration since the shark moved from northern to southern hemisphere, with more than 6600 km travelled, representing the longest migration ever recorded in a smooth hammerhead specimen.

### Vertical habitat use

**Fig 2:**
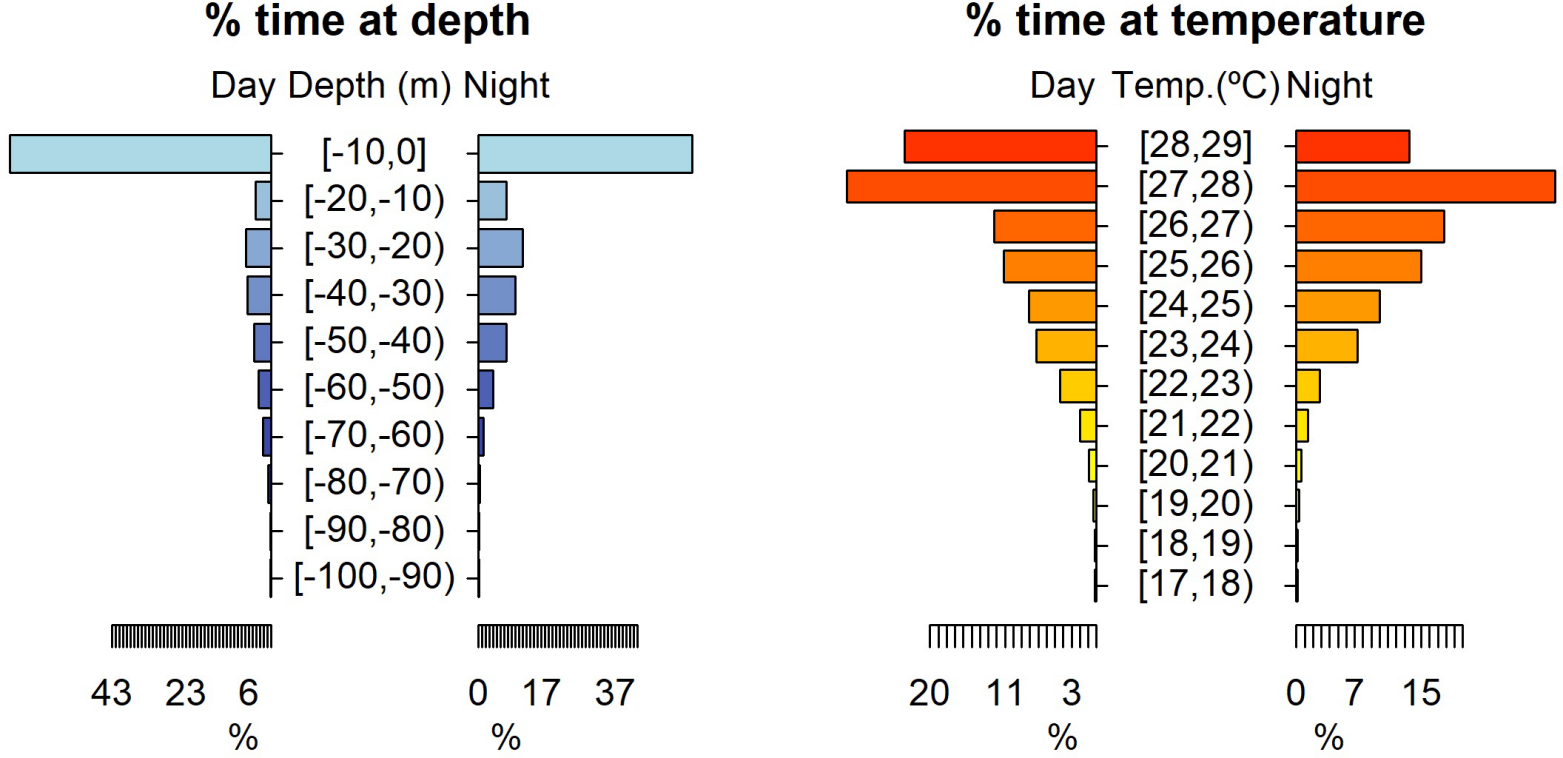
Habitat use for daytime and nighttime. Habitat use of smooth hammerhead shark, *Sphyrna zygaena*, for daytime and nighttime in terms of depth and temperature. Depth classes are categorized in 10 m intervals and temperature classes in 1°C intervals.

**Fig 3:**
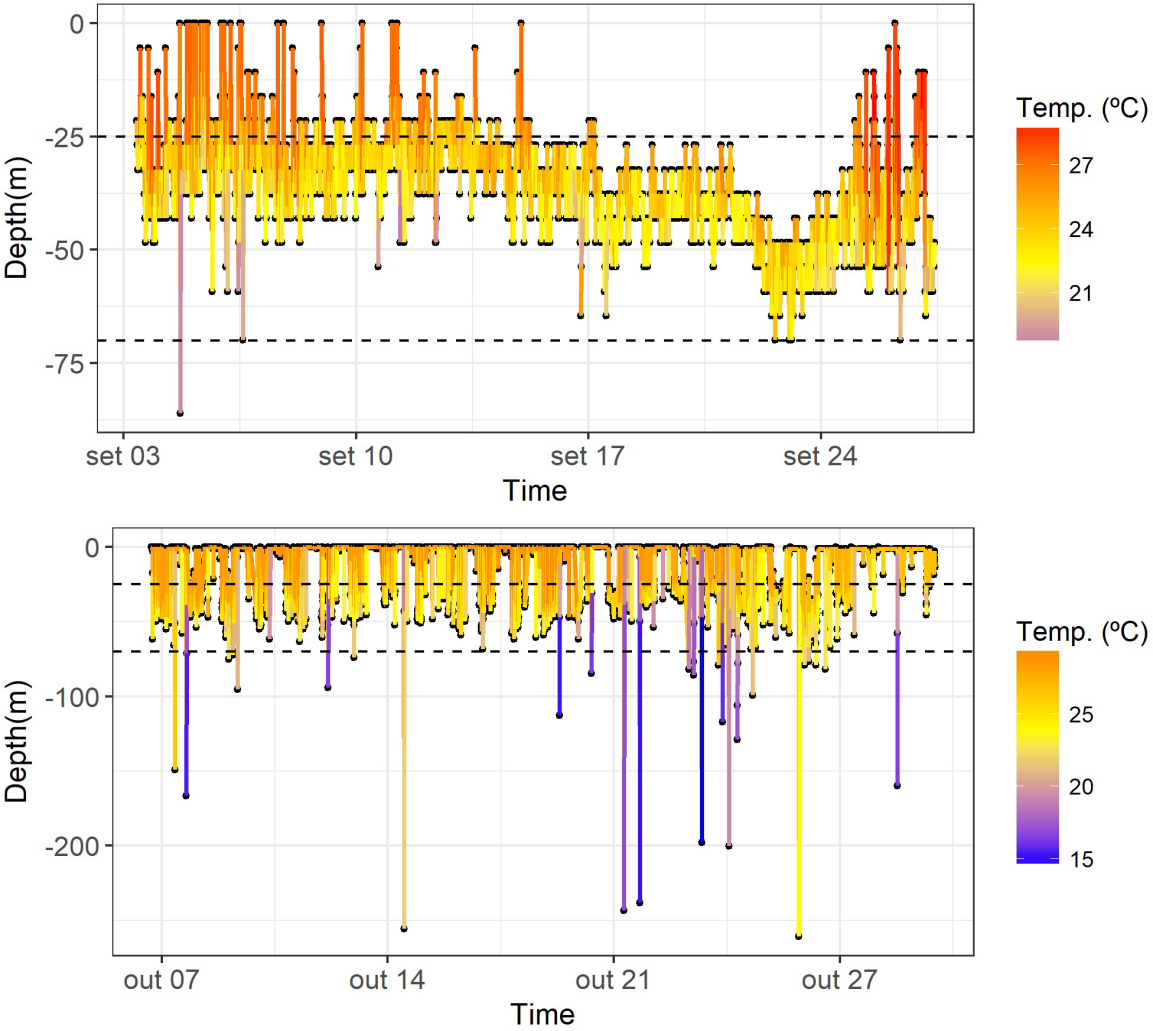
Diving behavior. Details of diving behavior profiles of smooth hammerhead sharks, *Sphyrna zygaena*, tagged with PSATs. The plot on the top (shark 113781) represents the most common behavior movements. The plot on the bottom (shark 127998) shows occasional deep dives. Horizontal dashed lines represent the 90% percentile depth distribution of the hooks (~25–70 m).

The vertical movements of the smooth hammerhead sharks did not exhibit any clear diel cyclicity. Although significant differences on habitat use between night and day were found (depth: permutation test differences = 3.19 m, p-value < 0.001; temperature: permutation test differences = -0.66°C, p-value < 0.001), sharks spent most of their time close to the surface (mean depth = 13.62 m, SD = 19.77 m; mean temperature = 26.28°C, SD = 2.06°C) within the depth-interval 0–50 m and preferred a warmer environment with water temperature above 23°C (Fig 2). Occasionally, deeper dives followed by rapid ascents were recorded. The maximum depth reached was 260.90 m and the minimum temperature recorded was 12.80°C (Fig 3).

**Fig 4:**
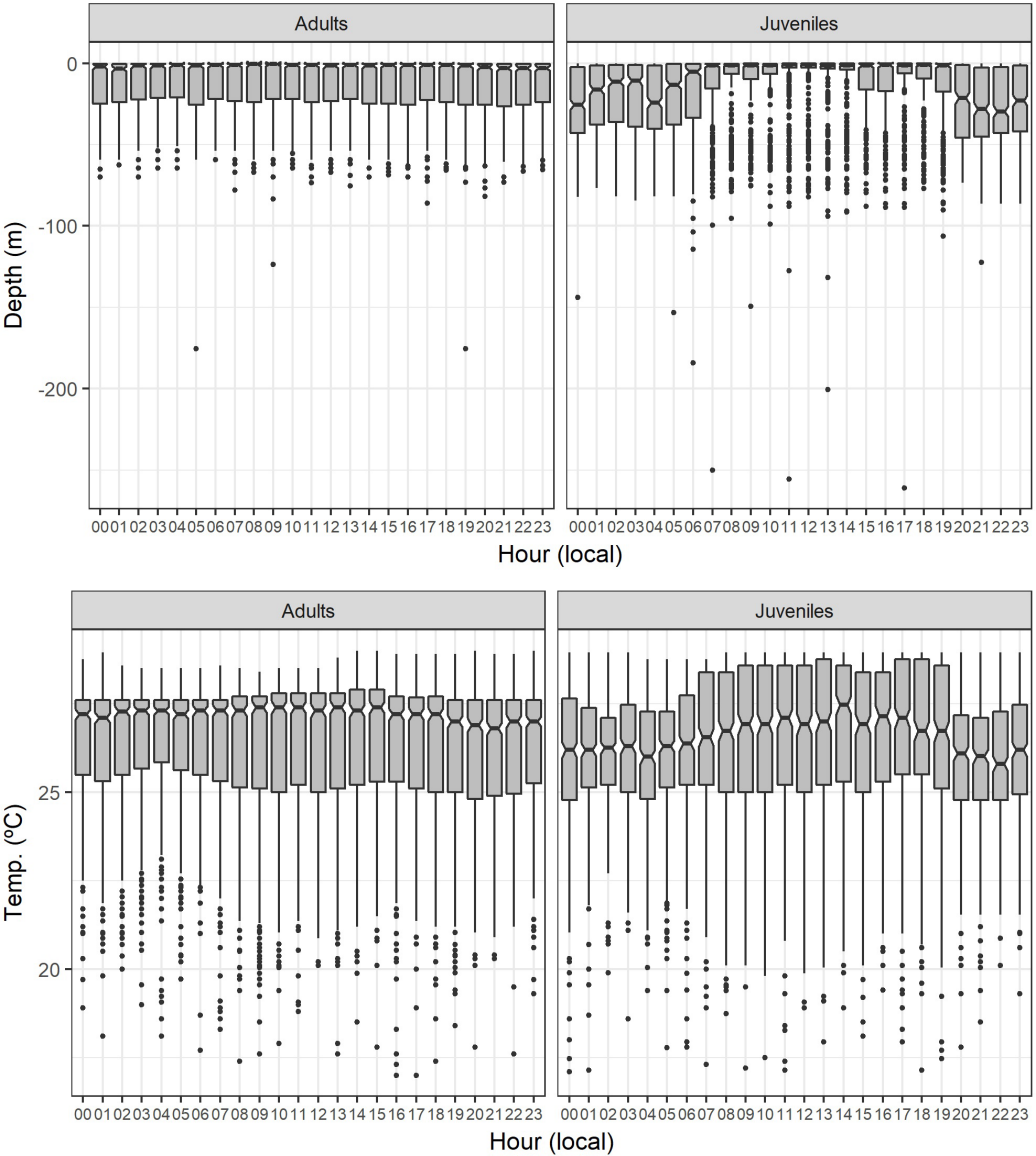
Habitat use per hour of day. Habitat use of smooth hammerhead shark, *Sphyrna zygaena*, with the data categorized in one-hour time classes, separated by maturity stage (adults and juveniles). The data represented is the median, the 1^st^ and 3^rd^ quartiles, the 95% confidence intervals of the median and the outliers.

Although both adults and juveniles showed a preference for shallow waters, different habitat use patterns were observed during daytime and nighttime. Juveniles occupied a wider range of vertical habitat than the adults, with the juveniles staying in deeper colder waters during the night (Fig 4). The mean depth during daytime was 10.99 m for adults and 13.72 m for juveniles (permutation tests: daytime differences = -2.73, p-value < 0.001), while during nighttime the mean depth was 11.23 m for adults and 21.81 m for juveniles (permutation tests: nighttime differences = -10.58, p-value < 0.001). Similar results were obtained for water temperature. The mean temperature during daytime was 26.43°C for adults and 26.28°C for juveniles (permutation tests: daytime differences = 0.15, p-value < 0.001), while during nighttime the mean temperature was 26.41°C for adults and 25.84°C for juveniles (permutation tests: nighttime differences = 0.57, p-value < 0.001).

**Fig 5:**
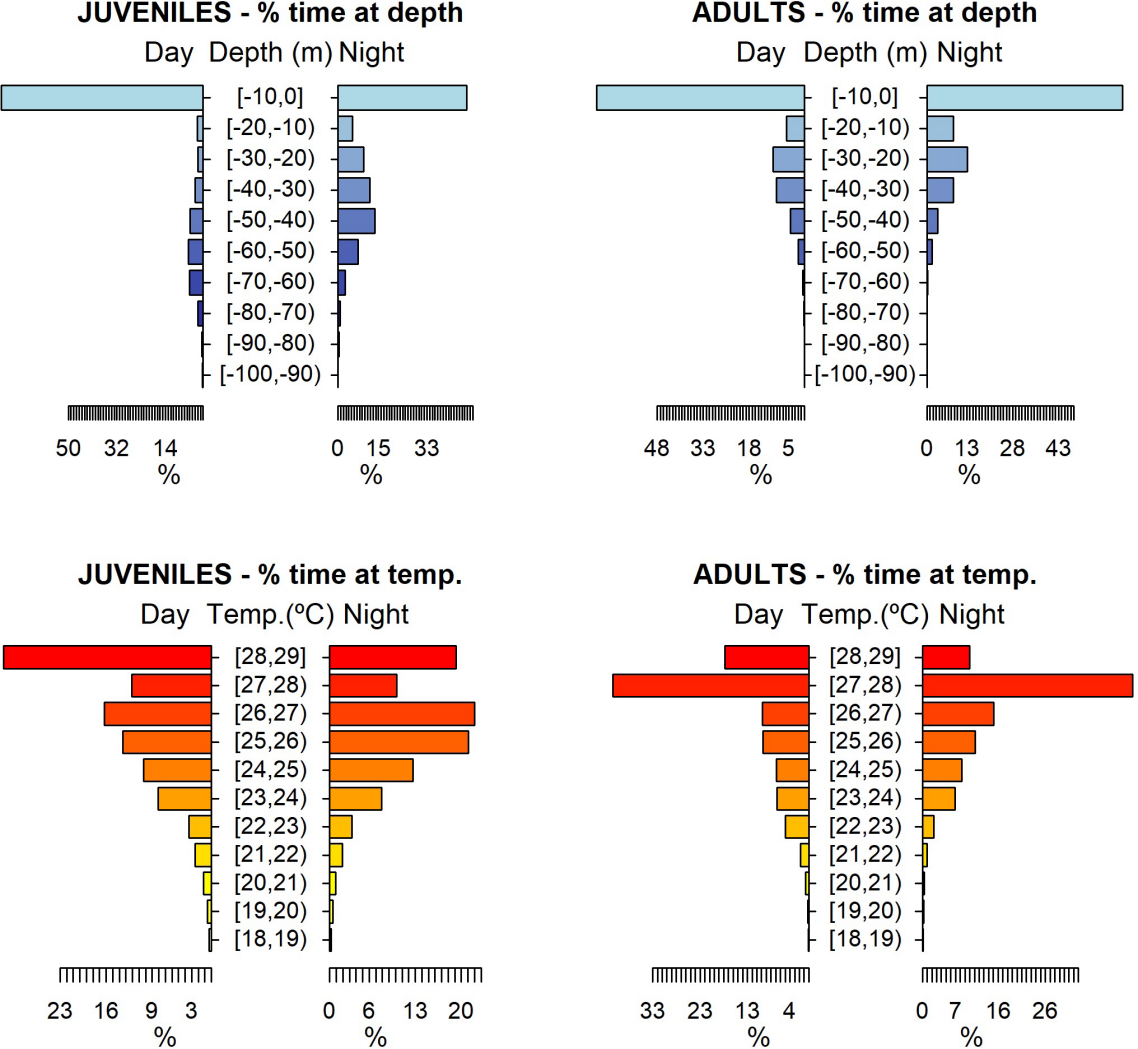
Habitat use per maturity state. Habitat use of smooth hammerhead shark, *Sphyrna zygaena*, per maturity stage (adults and juveniles), for daytime and nighttime in terms of depth and temperature. Depth classes are categorized in 10 m intervals and temperature classes in 1°C intervals.

Time-at-depth data revealed that the 0–10 m depth class was the most often occupied, independently of the time of day and the maturity stage of the sharks. The adults spent 67.86% and 63.86% of their day and nighttime, respectively, at 0–10 m, while the juveniles displayed peaks of 74.76% and 47.78% at such depth for day and nighttime, respectively (Fig 5). In addition, adults showed a preference for water layers of 27–28°C, with 41.45% and 44.44% of their day and nighttime, respectively, spent there. Juveniles preferred slightly warmer water temperatures (28–29°C) during daytime (31.62% of the daytime), whereas during nighttime the modal water temperature shifted to colder waters (26–27°C) where juveniles spent 22.03% of their nighttime (Fig 5).

### Overlap between shark habitat and fishing gear depth

The depth of hooks varied according to the length of the float lines. The average hook depth of the pelagic longline fishery was 41 m and 48 m, when using 12 m and 16 m float lines, respectively, with those differences statistically significant (Permutation test: observed differences = 6.68; p-value < 0.01).

**Fig 6:**
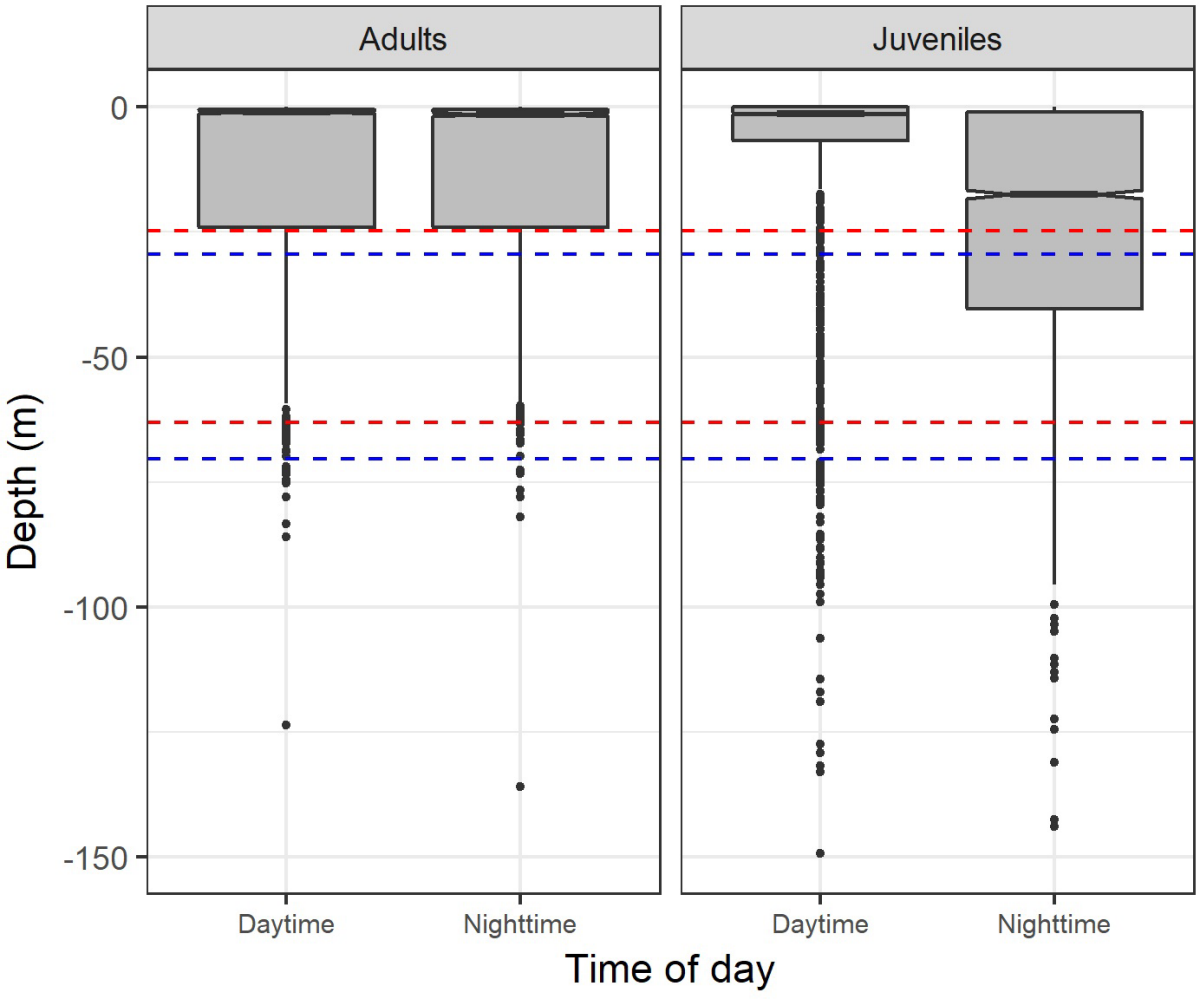
Overlap between the vertical habitat of smooth hammerhead shark and the hook depth distribution. Overlap between the vertical habitat of smooth hammerhead shark, *Sphyrna zygaena*, and the depth of operation of shallow water pelagic longlines targeting swordfish, separated by maturity stage (adults and juveniles), during daytime and nighttime. Horizontal dashed lines represent the 90% percentile depth distribution of the hooks for the 12 m (red lines) and 16 m (blue lines) float lines.

The 90% percentile depth distribution of the hooks was 24.8–63.1 m and 29.4–70.3 m for the 12 m and 16 m float lines, respectively. The analysis of the spatial overlap between hook depth distribution and the smooth hammerhead vertical habitat showed that there were no significant differences on the mean overlap time when comparing maturity stage (ANOVA: F = 2.231, p-value = 0.1502), daily period (ANOVA: F = 1.065, p-value = 0.3139) and length of the float lines (ANOVA: F = 0.032, p-value = 0.8593). However, there was a significant maturity stage-daily period interaction at a significance level of 10% (ANOVA: F = 3.334, p-value = 0.0821). The overlap was particularly noticeable for the juveniles during nighttime (Table 5) (Fig 6).

**Table 5:** Overlap between the vertical habitat of smooth hammerhead shark and the hook depth distribution.

Float line length	Daytime		Nighttime	
	Juveniles	Adults	Juveniles	Adults
12 m	16.4	21.5	41.2	21.7
16 m	18.6	16.1	37.1	14.2

Overlap, in percentage of time (%), between the vertical habitat of smooth hammerhead shark, *Sphyrna zygaena*, and the depth of operation of shallow water pelagic longlines targeting swordfish.

## Discussion

Understanding habitat preferences and vulnerability of smooth hammerhead sharks to fisheries is crucial to ensure successful species conservation strategies and effective management measures. The present work represents the most continuous recording of the movements and habitat use of smooth hammerhead sharks in the Atlantic Ocean. In this study, we were able to tag and track both juvenile and adult sharks, and the differences between maturity stages were analyzed and reported for the first time.

From the 8 tags deployed, only one failed to transmit, meaning the overall PSAT reporting rate was 88%. Four tags detached before their scheduled pop-up date, representing a premature release rate of 58%. However, this rate is lower in comparison with the average rate of 66% premature release reported by Hammerschlag et al. [49]. The causes of tag failure in data transmission and early tag detachment are still not well understood [26]. Battery failure, antenna damage, death of the tagged animal, biofouling or mechanical failure of the release mechanism have been reported as possible reasons of tag failure and early detachment [26, 50]. In addition, the success rate of PSATs also seems to be related with social behaviors of the tagged fish. Smooth hammerhead sharks have been observed swimming in schools [11, 51, 52], hence the touch of another animals swimming very close may trigger an early release by detaching the anchor, or even damage the PSAT.

It is important to mention that geolocation estimates have some limitations, since they are calculated from the ambient light-level irradiance which is influenced by variable natural conditions such as water clarity [25]. Shark diving behavior has also been described as an important factor while creating accurate estimates. Previous studies have documented some difficulties when using satellite technology to estimate geo-locations for deep-diving species [21, 53], as light attenuation with depth prevented the light-sensor on PSATs from correctly recording sunrise and sunset. However, the smooth hammerhead showed a preference for shallow waters both during the day and night, allowing us to use with higher confidence the recorded data for the horizontal spatial analysis.

Our findings showed that smooth hammerhead sharks moved in multiple directions over large areas and travelled long distances. Although the reasons for these horizontal movements are unknown, there are several aspects that might explain them. Two juvenile females were observed making excursions into inshore waters towards Cape Verde coast. Inshore waters are linked to increased shelter and food availability [54–56], and therefore may increase survival especially for juvenile sharks [57]. Thus, the movements towards inshore waters may be related to predator avoidance and/or foraging behavior. In addition, one adult female displayed the longest migration ever recorded for the smooth hammerhead shark (total distance travelled of 6610 km), which confirms the highly mobile nature of this species. Previous studies reported broad-scale horizontal movements for *S*. *zygaena* [27, 58], however this shark’s track represents the first trans-equatorial migration ever recorded and documented for this species. This movement might be driven by feeding events, since the shark moved towards the Benguela marine ecosystem, one of the most productive marine systems, which attracts many top predators including smooth hammerhead sharks [59]. Moreover, this movement may also be associated with water temperature. The specimen moved progressively into cooler waters (the cold Benguela Current), similarly to what has already been documented for smooth hammerheads, which are thought to undergo seasonal migrations towards cooler waters in the summer and warmer waters in the winter [11, 50]. With these results, it is interesting to highlight that the sharks did not travel to waters of the western Atlantic. This evidence may indicate the existence of separate western and eastern stocks in the Atlantic, contrary to the current north-south division used for all pelagic sharks by ICCAT [60]. When compared with the scalloped hammerhead [29–32], the smooth hammerhead seemed to be a more oceanic species.

Short dives below 100 m were sporadically observed during the study period, with no evidence of a diel activity pattern. In some cases, it was noted that there were deep dives with relatively warm waters even at depth around 200 m, while on some other cases the water temperature close to the surface was noticeable colder than expected. This may be related with eddies moving through the region, or other oceanographic features that could affect stratification. Although little is known about the function of these deep dives, they appear to be common in pelagic sharks (i.e. blue shark, scalloped hammerhead, bigeye thresher and oceanic whitetip [19, 21, 32, 34]). Foraging ecology has been suggested as the more likely explanation for deep diving behavior in sharks [61]. In swordfish, deep dives have also been described as feeding excursions targeting mesopelagic organisms in the deep scattering layer [60, 62]. In addition, as determined from a swimming behavior study by Klimley et al. [63], this type of movement may also be linked to orientation, since chemical and magnetic information are used to guide migrations.

Although preferring shallow waters (0–50 m) above 23°C, differences in the vertical habitat use were found when comparing maturity stages, with the juveniles staying in deeper colder waters than the adults during nighttime. Consequently, maturity stage differences resulted in differences in the chance of encounter with the pelagic longline fisheries for juveniles and adults. Our analysis of the overlap between the species vertical habitat use and the depth of operation of shallow setting longline fishing gear indicated it was more marked during nighttime (when the fishery operates) especially for the juveniles (~40% of overlap time), which is consistent with the distributional patterns observed for the smooth hammerhead shark. Thereby, juveniles are potentially more impacted than the adults by this particular fishery targeting mostly swordfish. However, information extracted from the Portuguese scientific observer database seems to contradict this theory, as of the 544 specimens captured between 2006 and 2015 in the tropics, only 38 (7%) were juveniles. One hypothesis for this observation may be related with the geographical overlap between the spatial and/or seasonal distribution of the population and the patterns of operation of the fishing fleets, in a way that the fleets are operating mainly in areas where adults are more predominant. Additionally, it is worth noting that the fact that hooks were baited (typically with squid or mackerel) was not considered in this analysis. Baits have attractant characteristics that may condition the behavioral responses of the fishes. A higher attraction of adults to the bait types used could also explain their higher predominance in the catches. A previous study by Coelho et al. [21] analyzed the overlap of fishing gear and habitat distribution of the bigeye thresher shark (*Alopias superciliosus*), and similar to what was observed in our study, the percentage of overlap time was greater for juveniles during nighttime (~60% of overlap time). Again, these results agree with those of Cortés et al. [12], who observed that smooth hammerhead was less vulnerable to pelagic longline fleets when compared to other pelagic sharks, including the bigeye thresher, mainly due to a lower overlap between the species habitat and fishing gear utilization.

The results presented in this study are a major contribution to increase the current knowledge on the smooth hammerhead ecology, habitat use in the pelagic environment, overlap and potential impacts with pelagic longline fishing gear. However, the sample size of seven transmitting tagged sharks is insufficient for deducing general conclusions. Long-term monitoring is required to better understand spatial distribution and habitat utilization patterns, namely in terms of sex-related differences. Future research on the smooth hammerhead shark should seek to identify hotspot areas for the species, including areas of concentration of juveniles, as well as mating/pupping grounds with the presence of the larger pregnant females. Nonetheless, the information presented here can be used to contribute to the provision of sustainable management tools such as mitigation measures to avoid unwanted sharks captures, as well as to serve as input for Ecological Risk Assessments for pelagic sharks caught in Atlantic pelagic longline fisheries.

## Supporting information

S1 Dataset Dataset considered for this study. (XLSX)
